# ﻿Redescription and geographical distribution of *Leiostracusobliquus* (Reeve, 1849) (Mollusca, Gastropoda, Simpulopsidae)

**DOI:** 10.3897/zookeys.1167.98707

**Published:** 2023-06-19

**Authors:** Maria Isabel P. F. Macedo, Ximena M. C. Ovando, Sthefane D’ávila

**Affiliations:** 1 Museu de Malacologia Prof. Maury Pinto de Oliveira, Instituto de Ciências Biológicas, Universidade Federal de Juiz de Fora, Rua José Lourenço Kelmer, s/n - Martelos, Juiz de Fora, 36036-330 Minas Gerais, Brazil Universidade Federal de Juiz de Fora Juiz de Fora Brazil; 2 Programa de Pós-graduação em Biodiversidade e Conservação da Natureza, Universidade Federal de Juiz de Fora, Minas Gerais, Brazil Universidade Federal de Juiz de Fora Juiz de Fora Brazil

**Keywords:** Anatomy, gastropods, land snails, Orthalicoidea, taxonomy

## Abstract

*Leiostracus* Albers, 1850 was established to allocate land snails from Central America and Northern South America. Currently, approximately 19 species are considered valid. However, for most of them, the internal morphology is unknown. *Leiostracusobliquus* was described as a species of *Bulimus* from the state of Bahia based on shell characters. Until now, knowledge of this species has been scarce. The discovery of ethanol-preserved specimens from MZSP of this species allowed us to characterize, for the first time, the internal anatomy of this species and update its distribution. The shell of *L.obliquus* has seven to eight whorls and a wide pale-pink disrupted band all over the teleoconch. The rachidian tooth is small rectangular, symmetric, with round edges, and without differentiated cusps. After comparing the anatomy and radular characters between the shells of *L.obliquus* and *L.carnavalescus*, we found remarkable similarities in the morphology and color pattern.

## ﻿Introduction

The land snails ascribed to the family SimpulopsidaeSchileyko 1999 are characterized by shells with simple aperture, without teeth and the absence of a penial sheath (Breure 1979; Schileyko 1999). Currently, this family includes three genera: *Rhinus* Albers, 1860, *Simpulopsis* Beck, 1837, and *Leiostracus* Albers, 1850, which are distributed in Central America, South America, and West Indies (Schileyko 1999). The taxonomy of Simpulopsidae has changed over time; its internal phylogeny and evolutionary relationship with other lineages of Orthalicoidea remain uncertain (Breure and Romero 2012). This taxon was first established by Schileyko (1999) as Simpulopsini, a tribe of Bulimulidae. Bouchet and Rocroi (2005) and Bouchet et al (2017) sustained this taxonomic status, but Breure and Romero (2012) raised this group to family rank, based on a three-locus gene phylogenetic analysis.

*Leiostracus* was first established to allocate land snails from Central America and Northern South America (Guyana, Suriname, and Brazil) (Albers 1850). The original diagnosis of this genus included shell characters, i.e., oblong-conic shell with 7–8 whorls, peristome expanded, and columellar margin dilated. Breure (1979) and Schileyko (1999) included internal anatomy traits in the diagnosis of this genus, i.e., short flagellum, absence of penial sheath, and spermathecal duct dilated at the base and narrow at the top.

The taxonomic history of *Leiostracus* is controversial, different authors allocate this taxon in generic or subgeneric ranks and ascribe it to different families over time (Albers and Martens 1860; Pilsbry 1899; Breure 1978). Pilsbry (1899) and Thiele (1929), considered *Leiostracus* as a subgenus of *Drymaeus* Albers, 1850, due to similarities in shell morphology. Pilsbry (1899), was the one who restricted the name *Leiostracus* to some species of *Drymaeus* endemic to Brazil, whereas Zilch (1959–1960), raised *Leiostracus* to the genus level. Breure (1979), also considered *Leiostracus* as a genus with geographic distribution restricted to Guyana, Suriname, and Brazil. There has yet to be a consensus on the number of valid species in this genus. Herein, Breure (1978), considered 15 number of species as valid. One year later, the same author raised the number of valid species to 39, and included in the subgenera, *Leiostracus* and *Pseudoxychona* Pilsbry, 1930, all endemic to Brazil (Breure 1979). Schileyko (1999), probably following Breure (1979), mentioned the same number of valid species of *Leiostracus*, while Salgado and Coelho (2003), considered only twelve valid species.

Currently, approximately 19 species of *Leiostracus* are considered valid (Simone 2006; MolluscaBase 2022), including three species described in the last decade: *Leiostracusfetidus* Salvador & Cavallari, 2014 (from Canavieiras, Bahia, Brazil), *Leiostracusfaerie* Salvador & Cavallari, 2014 (from Rio Doce, Espírito Santo, Brazil), and *Leiostracuscarnavalescus* Simone & Salvador, 2016 (from Nanuque, Minas Gerais, Brazil) (Salvador and Cavallari 2014a, 2014b; Simone and Salvador 2016). The available information on *Leiostracus* species is fragmentary and scarce. For most of them, the internal morphology is unknown. The anatomy of the soft parts is described only for *L.cinnamomeolineatus* Moricand, 1841, *L.demerarensis* (Pfeiffer, 1861) (Breure 1978; Muratov and Gargominy 2011), and *Leiostracuscarnavalescus* Simone & Salvador, 2016.

*Leiostracusobliquus* (Reeve, 1849), was initially described as a species of *Bulimus* Scopoli, 1777 from Bahia state, Brazil. Reeve (1849), did not mention the specific locality from where the type specimens were collected, and he established a diagnosis based entirely on the shell characteristics: “a pink shell of firm structure encircled with a conspicuous chestnut band around the last whorl” (sic.). So far, no other information besides the original description of this species is available. Dohrn (1883), ascribed *L.obliquus* to *Bulimulus* Leach, 1814, and designated six “varieties” based on the shell morphology and characteristics of the color bands, i.e.:

1. whitish without bands;
2. whitish, yellowish or reddish, with one band in the body whorl;
3. straw-yellow with rose broad bands and a brown band at the base of the body whorl or with a fine brown sutural line;
4. like the anterior but having a brown band around the umbilicus and two fine brown punctuated lines on the body whorl;
5. with four dark bands, the one upper the suture covering half of the whorls;
6. with four or five bands, the lowest around the umbilicus pale brown or dark, the other always dark, the uppermost and middle ones narrower and pale brown, the second lilac or rose, wider than the others, and frequently with a dark sutural line.

Dohrn (1883), also synonymized *Bulimusjeffreysi* Pfeiffer, 1852, with *L.obliquus*, because both species are similar in shell morphology. Ancey (1901), transferred *L.obliquus* to *Drymaeus* and named two varieties: Drymaeusobliquusvar.monozona Ancey, 1901 from Bahia and *D.obliquuspoecilogramma* Ancey, 1901 from Minas Gerais. Breure (1978), designed the lectotype based on similarities with the specimen drawn by Reeve (1849), in the original description and synonymized the variety *poecilogramma* with *L.obliquus*. After that, Breure (1979), considered this variety as subspecies of Leiostracus (Leiostracus), while for Simone (2006), the two subspecies established by Ancey were synonyms of *L.obliquus* while giving no clear justification. The designation proposed by Breure (1978) is currently accepted, and Leiostracus (Leiostracus) obliquus is ascribed to the family Simpulopsidae.

During a taxonomic revision of *Drymaeus* species from Brazil, while analyzing material from two Brazilian malacological collections from Museu de Zoologia de São Paulo and Museu de Malacologia Maury Pinto de Oliveira of Universidade Federal de Juiz de Fora, we found specimens labelled as “*Drymaeus*sp.” and we noticed that they corresponded to *Leiostracusobliquus*. Therefore, this work aims to redescribe *L.obliquus*, providing detailed information on the shell sculpture and internal anatomy. Furthermore, we assessed the geographic distribution of this poorly known species. We also compared the new data to the available information on closely related species.

## ﻿Materials and methods

### ﻿Morphology

Specimens were examined from the malacological collections of Museu de Zoologia de São Paulo (**MZSP**) and Museu de Malacologia Maury Pinto de Oliveira, Universidade Federal de Juiz de Fora (**CMMPO**). Images of the material deposited in the collections of the Academy of Natural Science of Philadelphia (**ANSP**) and the Zoological Museum of Amsterdam (**ZMA**) were also consulted. The species identification was based on the; shell morphology, original description of *L.obliquus*; and comparison with the images of the type material, besides the criteria proposed by Breure (1979) and the images provided by Simone (2006). Whole specimens examined were photographed using a Nikon D5300 digital SLR camera. Anatomical systems of ethanol-preserved specimens were dissected under a stereomicroscope Olympus SZX7, following the methodology proposed by Cuezzo (1997) and drawn with the help of a camera lucida. Measurements of the soft parts were expressed as a proportion of the length of the last whorl.

Radula and jaw were prepared for scanning electron microscopy, following the methodology described by Ploeger and Breure (1977). After being separated from the buccal mass, the radula and jaw were cleaned by immersion in a sodium hypochlorite solution, mounted on stubs, and metalized with 50 nm of gold. Details of the shell sculpture, jaw, and radula were obtained through scanning electron microscope FEI Quanta 250 at the Laboratório de Microscopia Eletrônica of the Universidade Federal de Juiz de Fora, Brazil (**UFJF**).

**Figure 1.**
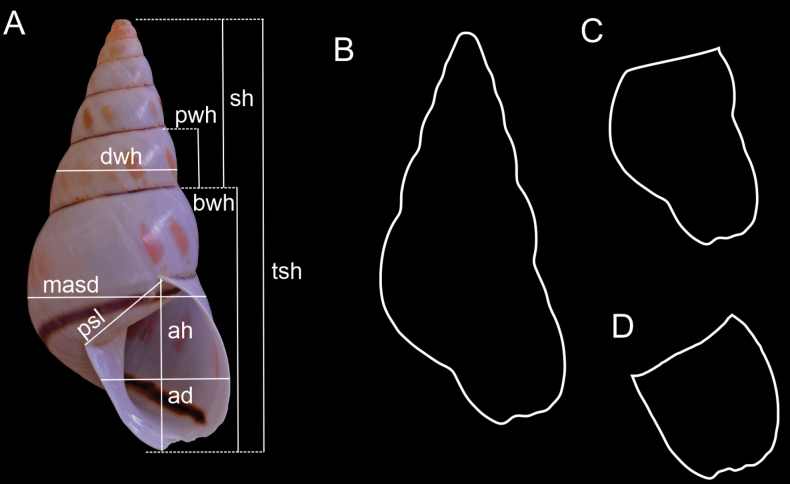
Measurements of the shell. **A** linear measurements **B** total shell perimeter **C** body whorl perimeter **D** aperture perimeter.

Digital images of specimens in standardized apertural view (aperture parallel to the camera lens) were used to create Tps files with TpsUtil v. 1.81 software (Rohlf 2021). Shell measurements were performed following the methodology proposed by Miranda (2015) and were obtained from these images with TpsDig2 v. 2.82 software (Rohlf 2018); i.e., linear measurements (Fig. 1A): total shell height (**tsh**), body whorl height (**bwh**), spire height (**sh**), major shell diameter (**masd**), minor shell diameter (**misd**), shell aperture height (**ah**), shell apertural diameter (**ad**), parietal space length (**psl**), penultimate whorl height (**pwh**), and penultimate whorl diameter (pwd) were obtained according to Miranda (2015). We also calculated the total shell area (**tsa**) and total shell perimeter (**tsp**) (Fig. 1B), spire perimeter (**sp**), body whorl perimeter (**bwp**) (Fig. 1C), and aperture perimeter (**ap**) (Fig. 1D), by drawing the shell outline directly from the images with Draw Curves tool from TpsDig2, which allowed computation of the enclosed area (cm^2^) and the perimeter (cm) of the outline.

### ﻿Occurrence records and distribution

To update the geographic distribution of *L.obliquus*, occurrence data were obtained from literature, and malacological collections consulted from the following sources: Global Biodiversity Information Facility (https://www.gbif.org), Sistema de Informação sobre a Biodiversidade Brasileira (https://www.sibbr.gov.br), the website of malacological collection of the Academy of Natural Science of Philadelphia (https://ansp.org). Despite the loss of the Museu Nacional do Rio de Janeiro’s material in the fire of 2018, the information associated with specimens from this collection remained available on biodiversity databases. We decided to include the occurrence records for *L.obliquus* from this collection even though we could not confirm species identification, as these specimens were originally identified by Dr. Norma Campos Salgado, a renowned Brazilian malacologist. After removing duplicate records and records without specific localities, we obtained a total of 40 occurrence records, which were georeferenced using Gazetteers and Geolocation applications (https://geo-locate.org/web/WebGeoref.aspx). Layers corresponding to Brazilian biomes and administrative boundaries were obtained from Instituto Brasileiro de Geografia e Estatística (IBGE, https://www.ibge.gov.br). Distribution maps were constructed using QGis version 3.10.14-A Coruña (QGIS Development Team 2021).

### ﻿Institutional acronyms

**MCZ**Museum of Comparative Zoology, Harvard University, USA;

**MNRJ** Museu Nacional do Rio de Janeiro, Rio de Janeiro, Brazil;

**MZSP** Museu de Zoologia de São Paulo da Universidade de São Paulo, São Paulo, Brazil;

**NHMUK**Natural History Museum, London, UK.

## ﻿Results

### ﻿Taxonomy

**Family Simpulopsidae Schileyko,1999**

#### ﻿Genus Leiostracus

Taxon classificationAnimaliaStylommatophoraSimpulopsidae

Albers, 1850

947D2364-366D-5CB6-B230-D1A544F42A13

##### Type species.

*Leiostracusvittatus* (Spix, 1827) by subsequent designation.

#### ﻿ Leiostracus obliquus

Taxon classificationAnimaliaStylommatophoraSimpulopsidae

(Reeve, 1849)

7AB396BA-ABE8-5CAB-9021-58D954C06957

**Figure 2.**
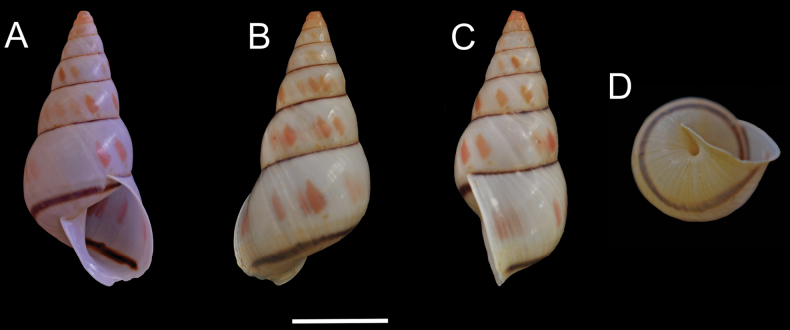
*Leiostracusobliquus* (Reeve, 1849). Specimen from Mantena, Minas Gerais (MZSP 43185) **A** apertural view **B** dorsal view **C** lateral view **D** periumbilical region. Scale bar: 1 cm.

**Figure 3.**
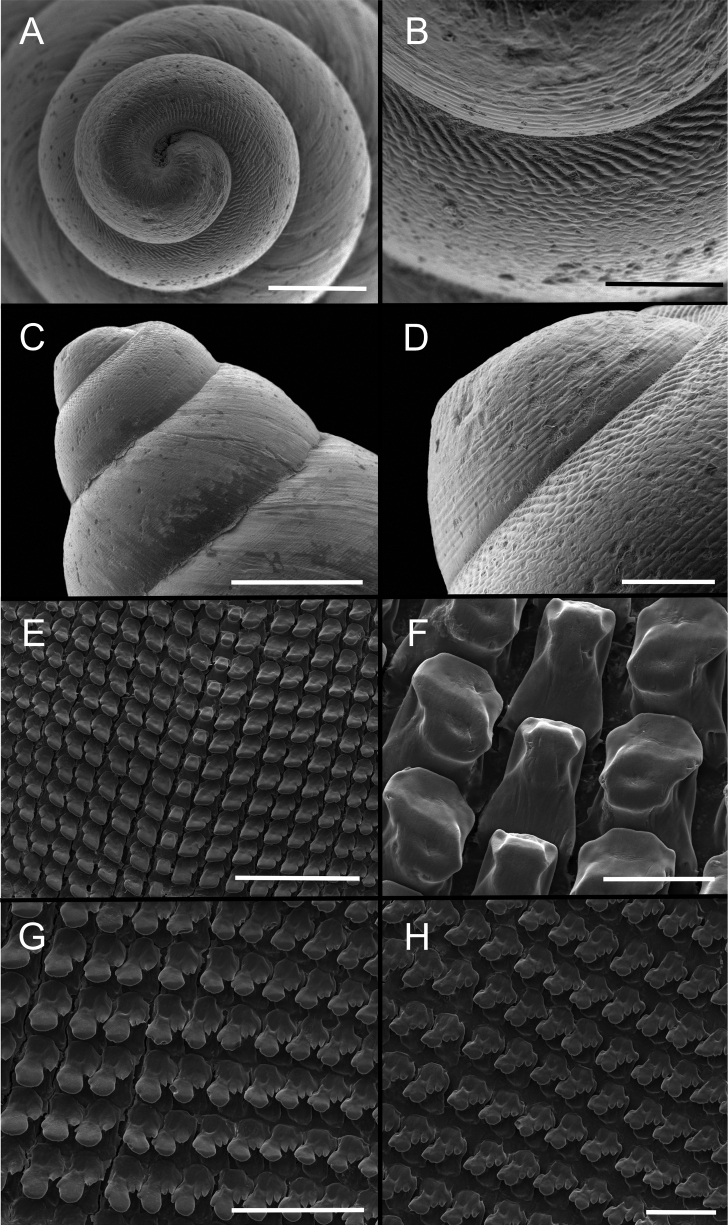
Images of protoconch and radula of *Leiostracusobliquus* (Reeve, 1849) scanning electron micrograph (SEM) **A** protoconch showing sculpture **B** detail of sculpture of the second whorl showing the two sculpture types, first whorl with regular striation and second whorl with oblique irregular striations in the upper part, in the middle and lower part with punctuation aspects **C** general view of the first tree whorls **D** general view of the protoconch and first two whorls **E** general view of the radula showing the teeth arrangement **F** detail of rachidian teeth and first lateral teeth **G** detail of eighth and ninth lateral teeth (arrow) showing the transition from lateral to marginal teeth **H** detail of lateral region. Scale bars: 75 μm (**A**); 250 μm (**B**); 100 μm (**C, G**); 1 µm (**D**); 200 μm (**E**); 40 μm (**F**); 50 μm (**H**).

**Figure 4.**
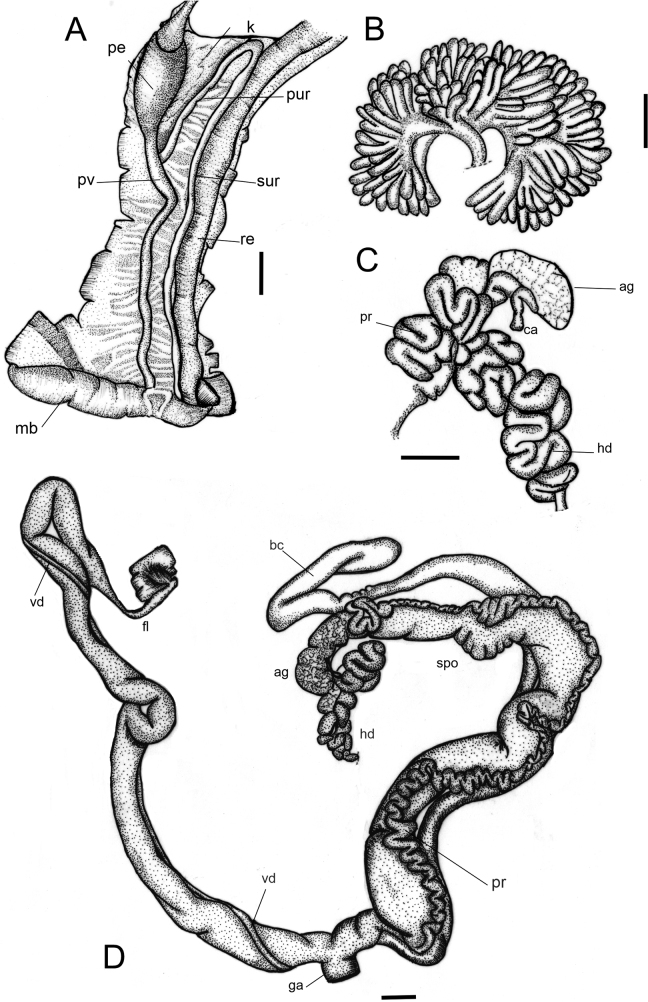
*Leiostracusobliquus* (Reeve, 1849), anatomy **A** ventral view of the pallialsystem dissected out, showing the inner face of ventral view **B** detail of albumen gland showing internal lobs **C** ventral view of the reproductivesystem without ovotestis **D** dorsal view of the reproductivesystem. Abbreviations: ag, albumen gland; bc, bursa copulatrix; ca, carrefour; fl, flagellum; ga, genital atrium; hd, hermaphrodite duct; k, kidney; mb, mantle border; pe, pericardium; pr, prostate; pur, pulmonary roof; pv, pulmonary vein; re, rectum; spo, spermoviduct; sur, secondary ureter; vd, vas deferens; ur, ureter. Scale bar: 2 mm (**A–D)**.

Figs 2 , 3 , 4 , 6A–C

Bulimus obliquus Reeve (1849) [1848–1850]: 148; — Pfeiffer (1853): 342.
Bulimulus obliquus —Dohrn (1883): 352; —Martens (1885): 191.
Bulimus jeffreysi Pfeiffer (1852): 93; —Pfeiffer (1853): 342. — Pilsbry (1899) [1898–1899]: 93.
Drymaeus obliquus — Ancey (1901): 93; —Pilsbry (1899) [1898–1899]: 93, pl. 14 fig. 14.
Drymaeus obliquus var. monozona Ancey, (1901): 93.
Drymaeus obliquus var. poecilogramma — Ancey (1901): 93.
Leiostracus obliquus — Breure (1979): 127; —Salgado and Coelho (2003): 162; —Simone (2006): 122; —Breure and Ablett (2015): 37.
Leiostracus (Leiostracus) obliquus — Breure 1978: 227 [lectotype designation].

##### Type material.

***Lectotype*.** Brazil • 1 shell; NHMUK 1975493. ***Syntype*** of *B.jeffreysi*: Brazil • 3 shells; NHMUK 20110083.

##### Type locality.

“Bahia” (Reeve 1849).

##### Original diagnosis.

“*Bul testâ subpyramudali-ovatá*, *umbilicatá*, *crassiusculá*, *ad basin oblique product*, *anfractibus septen ad octo*, *lævibus*, *aperturá obliquá*, *columella labroque latè dilatates*; *pallidè rosacea*, *anfracta ultimo fascia castaneâ unicâ cingulato.*” (Reeve 1849)

##### Material examined.

Brazil • 9 specimens preserved in ethanol 70%; Minas Gerais, Mantena; Coltro leg.; MZSP 43185 • 1 shell; Espírito Santo, Itapemirim; 22 Apr. 2001; Castro, G.A. leg.; CMMPO 8795 • 16 shells; Espírito Santo, Itapemirim; Mar. 2001; Castro, G.A. leg.; CMMPO 8797.

##### Museum material.

Brazil • 1 shell; Minas Gerais; Vanatta, E. G. leg.; ANSP 79491 • 1 shell; Espírito Santo, South of Guarapari, Meaípe; Dec. 1992; Bodart, A. leg.; ANSP 426466 • 4 shells; Espírito Santo, near Posta de Souza, Doce River; MCZ 57073 • 6 shells; Espírito Santo, Santa Teresa, Santa Lúcia, Trilha do Araponga; 22 Jan. 2002; MNRJ 9565 • 7 shells; Espírito Santo, Cachoeiro do Itapemirim, Cafundó, Mata do Panelão; 20°48'16"S, 41°09'31"W; 22 Mar. 2002; MNRJ 9566 • 4 shells; Espírito Santo; Cachoeira do Itapemirim, Fazenda Cafundó, trilha 1; 20 Jan. 2001; MNRJ 9562 • 1 shell; Espírito Santo, Linhares; Oct. 1973; MNRJ 9568 • 1 shell; Espírito Santo, Linhares; 10 Nov. 1973; Salgado, N. C. leg.; MNRJ 33330 • 6 shells; Espírito Santo, Linhares; Sep. 1972; MNRJ 9569 • 6 shells; Espírito Santo, Linhares; Sep. 1972; MNRJ 9563 • 12 shells; Espírito Santo, Linhares; Sep. 1972; MNRJ 9564 • 1 shell; Espírito Santo, Linhares; Nov. 1972; MNRJ 9567 • 2 shells; Espírito Santo, Itaguaçú; Sep. 1971; MNRJ 9606 • 4 shells; Espírito Santo, Baixo Grande; Oct. 1971; Salgado, N. C. leg.; MNRJ 32846 • 6 shells; Espírito Santo, Baixo Guandú; MNRJ 9604 • 1 shell; Espírito Santo, Pedro Canário, Rio Itaunas, Faz. Rochysky; Jun. 1970; MNRJ 8332 • 2 shells; Rio de Janeiro; Salgado, N. C. leg.; MNRJ 31149.

##### Redescription.

***Shell*** (Figs 2A–D, 3). Shell perforate, pyramidally ovate, umbilicated, thick. Shell height from 21.87 to 30.81 mm (Table 1); seven or eight whorls slightly convex. Protoconch 1½ whorls, white to pale pink, with well-marked regular striation (Fig. 3A). Teleoconch sculptured with delicate spiral striae (Fig. 3C), with a single narrow dark-brown line in the last whorl, surface with regular and shallow striae. Spire high, ~ 3/5 of the shell length. Second whorl with two forms of sculpture, oblique irregular striations in the upper part, irregular striations in the middle part, and spiral lines, with punctuations in the lower part (Fig. 3B–D). The transition between protoconch and teleoconch well marked. Aperture broad, oblique, ovate to subovate. Outer lip and columella broadly reflected (Fig. 2A). Suture well-marked, simple, slightly oblique, with a thin purple-brown line (Fig. 2B). Body whorl, white to pale cream; wide pale-pink disrupted band all over the teleoconch (Fig. 2A–C). Peristome widely expanded in adult specimens (Fig. 2C). Body whorl ~ 2/5 of the shell length, rounded; umbilicus narrow, partially covered by the peristome; periumbilical area pale cream (Fig. 2D).

**Table 1.** Average, range, and ratios of measurements (mm) from shells of *Leiostracusobliquus* for material housed in Museums. ad: shell apertural diameter; ah: shell apertural height; bwh: body whorl height; masd, major diameter of the shell; misd: minor diameter; psl: parietal space length; pwd: penultimate whorl major diameter; pwh: penultimate whorl height; sh: spire height; tsh: shell height). Shell perimeter (mm) (sp: shell perimeter; ssa: shell surface area; spp: spire perimeter; spsa: spire surface area; bwp: body whorl perimeter; bwsa: body whorl surface area; ap: aperture perimeter; asa: aperture surface area).

Measurement	*Leiostracusobliquus* from mantena *n* = 9	Lectotype NHMUK 1975493	ANSP 79491	ANSP 426466
**tsh**	30.81-21.87 (±2.75) 24.52	22.7	22.51	23.23
**bwh**	19.08-14.08 (±1.57) 15.26	12.58	14.43	13.24
**sh**	11-73-7.71 (±1.3) 9.39	7.96	8.39	10.03
**masd**	14.06-10.02 (±1.2) 11.48	12.05	9.88	10.25
**misd**	10.24-8.94 (±0.52) 9.52	7.99	9.01	9.08
**ah**	12.84-9.06 (±1.12) 10.06	9.45	9.91	9.31
**ad**	9.08-5.09 (±1.15) 6.98	6.97	7.04	7.48
**psl**	7-19-4.88 (±0.93) 5.97	6.44	6.49	6.74
**pwh**	4.07-2.99 (±0.39) 3.37	3.10	3.11	3.86
**pwd**	7.88-6.67 (±0.39) 3.37	6.63	6.85	7.49
**sp**	65.1-52.2 (±0.44) 56.9	47.99	54.87	56.68
**ssa**	212.0-151.4 (±22.6) 170.3	127.99	164.11	168.11
**spp**	34.6-17.5 (±4.9) 29.2	24.95	27.04	30.29
**spsa**	68.2-32.9 (±11) 45.4	34.43	37.96	48.12
**bwp**	48.8-42 (±2.56) 44.1	37.5	43.06	42.36
**bwsa**	168.2-109.5 (±18.9) 127.8	93.21	125.13	119.86
**ap**	33.5-25.6 (±2.6) 28.8	26.36	27.72	27
**asa**	66.2-25 (±12.2) 44.6	47.94	49.46	50.54

***Radula*** (Fig. 3F–H). Approximately 75 teeth per row. Mean number of teeth per half row (except for central tooth) 37. Each radular row disposed linearly on the same level (Fig. 3E). Rachidian tooth small (~ 4/5 of the length of laterals), rectangular, symmetric, with round edges, without differentiated cusps (Fig. 3F); basal plate almost trapezoidal; cutting edge slightly round. Lateral teeth asymmetrical, bicuspid, basal plate wider, weakly arched towards the median region, and with cutting edge, ½ the size of the rachidian tooth (Fig. 3G). First lateral tooth with conspicuous inner cuspid; base wider than the apical part. The size of the lateral teeth decreases gradually towards the outer area. Basal-lateral cusp appears in the third or fourth teeth and becomes more distinct in the marginal teeth. Marginal teeth with four cuspids, mesocone well developed, with a bicuspid cutting edge, endocone relatively reduced, bicuspid or tricuspid (Fig. 3H).

***Pallial system*** (Fig. 4A). Mantle border simple, thick. Rectum narrow, walls thick. Primary ureter along the rectal side of the kidney up to the top of the lung cavity. Secondary ureter, wide and slender, parallel along the entire left side of the rectum. Mantle cavity short, ~ 1/4 of the body whorl. Kidney elongated, narrowly triangular, thin, ~ 1/3 of the pallial cavity in length, with ~ 7 lobes, the anterior lobe at the level of the auricle slightly longer. Pericardial cavity on the left side of the kidney, at the columellar margin of the posterior end of the pallial cavity, near the same length as the kidney. Auricle elongate, ~ 1/3 of the triangular ventricle. Pulmonary vein visible in the mantle roof, almost as long as the pallial cavity, oblique in the anterior 1/2, converging to the pneumostome region along with the ureter and rectum. Slender vessels visible on both sides of the pulmonary vein, at the distal portion of the pallial system.

***Genital system*** (Fig. 4B–D). Ovotestis with three or four branches of enlarged digitiform acini, each branch with more than 20 simple acini. Hermaphroditic duct tubular, very sinuous, thick, folds itself in an S-shape. Albumen gland small, slightly elongated (Fig. 4B, C). Spermoviduct, bursa copulatrix and penial complex with approximately the same length when distended. Bursa copulatrix elongated (~ 1/3 larger than spermoviduct), thin. Bursa sac slightly elongated terminally, lying on the concave face of the spermoviduct. Prostate long, with numerous tubular-acini, in the columellar surface of the spermoviduct. Penial complex long, club-shaped, phallus subcylindrical, penis with a sheath. Phallus-epiphallus transition unrecognizable externally. Insertion of the vas deferens subterminal. Flagellum present, subcylindrical, short, ~ 1/10 of penial complex length. Insertion of the bursa copulatrix and penial complex into the free oviduct at the same level. Genital atrium very short, ~ 1/10 of spermoviduct length. Spermatheca well-differentiated, sinuous, lying at the superior third of the albumen gland.

##### Distribution.

**Figure 5.**
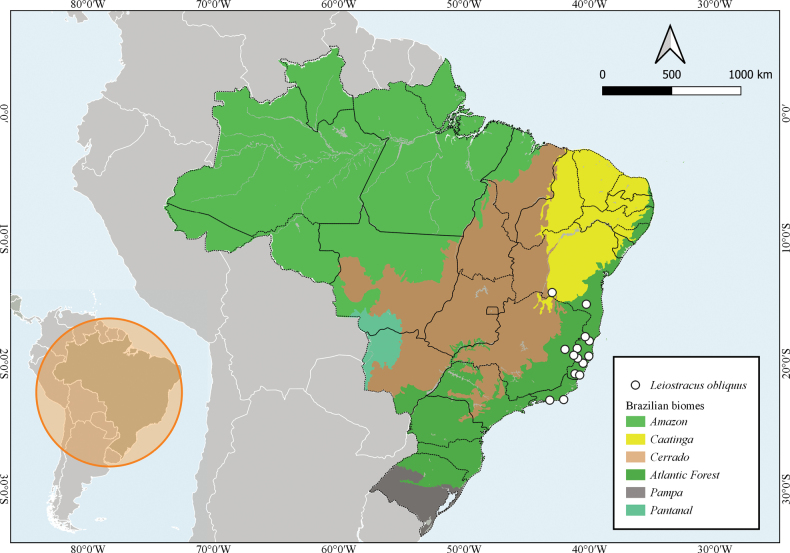
Geographical distribution of *Leiostracusobliquus* (Reeve, 1849). Occurrence records are marked with white circles. Lower left margin: administrative map of South America, showing Brazil.

Brazil. Bahia, Espírito Santo, Minas Gerais, Rio de Janeiro, and São Paulo states (Suppl. material 1). Localities of occurrence are mainly in the Atlantic Forest biome (Fig. 5).

##### Remarks.

**Figure 6.**
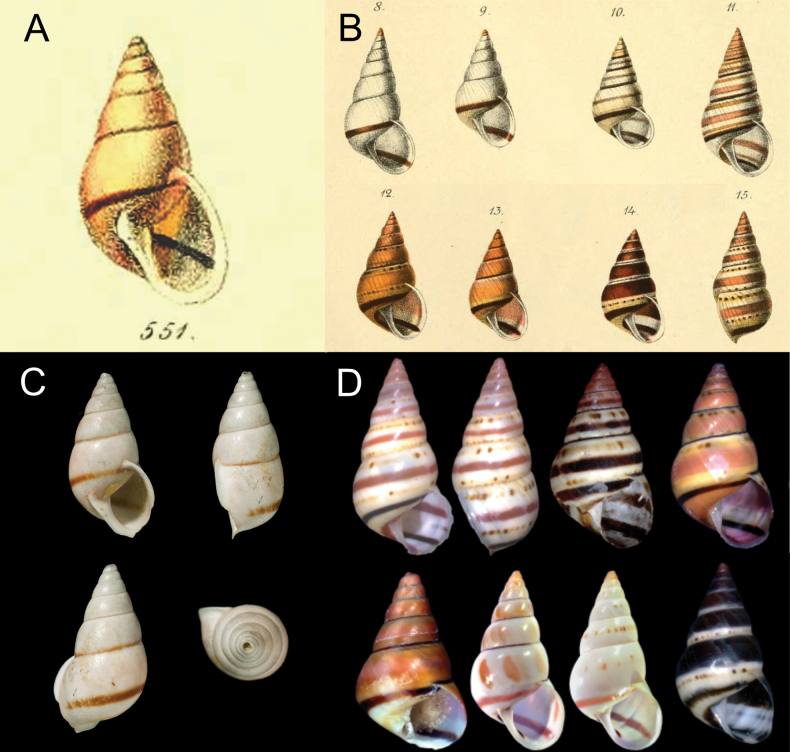
Variation on color pattern in *Leiostracusobliquus* (Reeve, 1849). **A** painting from the original description of *L.obliquus* in Reeve (1849)**B** varieties from Minas Gerais described from Dohrn (1883)**C***Leiostracusobliquus* Lectotype (NHMUK 1975493) **D***Leiostracuscarnavalescus* varieties from Simone and Salvador (2016) with permission.

The shell characters of the specimens from MZSP, identified here as *L.obliquus* matched with the original description made by Reeve (1849) and the lectotype designed by Breure (1978) (Fig. 6A, C, Table 1). After comparing our specimens with the material from several malacological collections, we concluded that *L.obliquus* is very similar to *Leiostracuscarnavalescus* Simone & Salvador, 2016. Both species are medium-sized (24.5 mm ± 2.7 in *L.obliquus* whereas in *L.carnavalescus* 24.4 mm ± 1.3) (Table 1), presenting conical-oval shells, with a typical brown line on the base of the body whorl (Fig. 6A), 7–8 whorls, and outer lip widely reflected. In the original description of *L.carnavalescus*, Simone and Salvador (2016) provided two images of a specimen from Mantena, whose shell characteristics match the original description of *L.obliquus*. We also observed notable similarities between Dorhn’s “varieties” and the three color patterns described for *L.carnavalescus*, all of them exhibiting a brown band on the base of the body whorl mentioned by Dohrn (1833) and Pilsbry (1899) for *L.obliquus* (Fig. 6B, D). The holotype of *L.carnavalescus* is identical to Dorhn’s “variety 2” (Fig. 6B; Dohrn 1833: fig. 11). The paratypes #1 and #2 (MZSP 106179 and M`ZSP 106178, respectively) of *L.carnavalescus* are similar to Dorhn’s “variety 4”, presenting pinkish bands and fine lines of brown spots (Fig. 6B; Dohrn 1833 date: fig. 15). Dorhn’s “variety 3” (Fig. 6B; Dohrn 1833: fig. 13) is similar to “specimen #2” of *L.carnavalescus* from Sooretama (Fig. 6D), both present yellowish shells, with wide pinkish bands, a brown band at the base of the body whorl, and a thin brown sutural line. Lastly, Dorhn’s “variety 5” (Fig. 6B; Dohrn 1833: fig. 14) is similar to the paratype #3 (MZSP 106179) of *L.carnavalescus*. Both presents wide dark bands with a fine brown line of punctations. Dohrn (1883) did not specify where the material analyzed by him was deposited. Nonetheless, we found two lots donated by Dorhn and identified as *L.obliquus* from Brazil, housed in the Zoölogisch Museum of Amsterdam (ZMA 385620; ZMA 385621). We believe this material could be part of the specimens used by Dorhn to establish those color varieties of *L.obliquus*. The shell from ZMA 385620 is similar to Dorhn’s “variety 3”, while the shells from ZMA 385621 are similar to Dorhn’s “variety 1” and “variety 2”. The analysis of these specimens also confirmed the similarities between *L.obliquus* and *L.carnavalescus*. The radulae of both species are identical: the rachidian tooth is rectangular, symmetric, without differentiated cusps and the basal plate is almost trapezoidal; cutting edge slightly round; lateral teeth bicuspid and weakly arched towards the median region (Table 2). The anatomy of the reproductivesystem is also identical in both species (mainly the presence of the penis sheath and the morphology of the penis complex).

**Table 2.** Comparison between *Leiostracusobliquus* and other species of *Leiostracus* that occur in Brazil. Abbreviations: H: total shell height; D: Major diameter of the shell; h: aperture height. The marking * indicates that the internal anatomy from a rehydrated specimen.

Species	Type locality	Number of whorls	Shell measurements	Radula	Reproductivesystem	Distribution
*L.obliquus* (Reeve, 1849)	Brazil, Bahia	7-8	H: 23 mm D: ~12.7 mm h: ~11.5 mm	Rachidian, symmetric, rectangular. Laterals: bicuspid. Marginals: tetracuspids.	Penis with penis sheath. Flagellum short, subcylindrical. Spermatheca reservoir claviform.	Brazil (Bahia, Espírito Santo, Minas Gerais, Rio de Janeiro, São Paulo).
*L.carnavalescus* Simone & Salvador, 2016	Brazil, Minas Gerais, Nanuque	8	H: ~25 mm D: 12.4 mm h: 9.2 mm	Rachidian: triangular; Lateral and marginals: asymmetrical similar to rachidian.	Penis with penis sheath. Flagellum absent. Spermatheca reservoir elliptical.	Brazil (Espírito Santo, Minas Gerais)
*L.cinnamomeolineatus* (Moricandi, 1841)	"*Habitat la Province de Bahia*"	6 2/3	H: 21.5 mm D: 10.17 mm h: 8.58 mm	Rachidian: triangular; Laterals: ectocone reduced; Marginals: asymmetric, tricuspids.	Penis without sheath. Flagellum short, slender, U-shaped. Spermatheca reservoir globose.	Brazil (Bahia, Espírito Santo, Pernambuco).
*L.demerarensis* (Pfeiffer, 1861)	Guyana, Demerara	6.1	H: 17.95–20.64 mm D: 10.66 mm	Rachidian: monocuspid Laterals: bicuspid Marginals: weakly tricuspid.	Penis with penis sheath. Spermatheca reservoir small, long duct.*	Guyana; Surinam; Brazil (Maranhão, Pará).
*L.faerie* (Salvador & Cavallari, 2014)	Brazil, Espírito Santo, area in vicinity of Doce River	6	H: 14.6 mm D: 7.6 mm h: 5.8 mm	Without description	Without description	Kown only from type locality.
*L.fetidus* Salvador & Cavallari, 2014	Brazil, Bahia, Canavieira	6	H: 21.2 mm D: 10.5 mm h: 9.4 mm	Without description	Without description	Kown only from type locality.

On the other hand, *L obliquus* differs from *L.cinnamomeolineatus* in the morphology of the radula: in *L.obliquus* the rachidian tooth is nearly round, while in *L.cinnamomeolineatus* it is triangular (Table 2). However, this variation may be the result of age and/or diet. Considering the anatomy of the reproductivesystem, both species can be differentiated by the shape of the flagellum, which is longer and slender in *L.obliquus*, and inverted U-shaped and less tapered flagellum in *L.cinnamomeolineatus*, as well as by the shape of the bursa copulatrix reservoir, which is claviform in *L.obliquus* and globose in *L.cinnamomeolineatus* (Table 2).

## ﻿Discussion

In the present work, we redescribe the species *Leiostracusobliquus* (Reeve, 1849), providing new information on shell micro sculpture, radular morphology, and anatomy of the soft parts. The striking similarities in shell morphology and color pattern between *Leiostracusobliquus* and *L.carnavalescus*, evidenced by comparison with the type material, the species original descriptions, and other specimens from malacological collections leads us to think that these are the same species. Notwithstanding, DNA analyses comparing *L.obliquus* specimens with *L.carnavalescus* type material are necessary to clarify these assumptions.

The material from Zoölogisch Museum of Amsterdam was donated by Dorhn and probably was used by him to describe varieties of *L.obliquus* (Dohrn, 1883) which are identical to the three color patterns described for *L.carnavalescus*. Moreover, the specimens herein identified as *L.obliquus* cannot be differentiated from *L.carnavalescus* through the anatomy of the soft parts and radular morphology.

*Leiostracusobliquus* was described from a single dry shell and since its description, records of this species are scant and outdated. *L.obliquus* is mentioned in the most recent Brazilian species lists by Salgado and Coelho (2003) and Simone (2006), but there are no more recent occurrences. Although most species of the genus have been documented for Bahia, *Leiostracusvittatus* (Spix, 1827) was the sole species of the genus that was reported in the inventory of Northeastern Brazil from Dutra-Clarcke and Souza (1991). Since then, records of this species have been virtually nonexistent, highlighting the importance of native species inventories, particularly, for imperiled areas such as Atlantic Forest.

Comparisons between the shells of the varieties from *L.obliquus* and *L.carnavalescus* showed remarkable similarities in morphology and color pattern. Concerning the “specimen #1” (MZSP 108010) from Mantena, Simone and Salvador (2016) mentioned: “…seem to belong to the same species as far as the conchological characters indicate.” This matched well with our material of *L.obliquus*, reinforcing the similarity of both species. The “variety 2” corresponds with the drawn specimen from the original description (Reeve 1849) and would be the typical form of *L.obliquus*, while the other varieties described by Dornh are identical to the shell color pattern mentioned by Simone and Salvador (2016). Furthermore, both species cannot be distinguished by their radula and reproductivesystem.

Color polymorphism refers to the occurrence of multiple discrete color phenotypes within the populations of the same species (White and Kemp 2016). The occurrence of color polymorphism is widespread in animals and usually has a genetic basis (Estévez et al. 2020). It may be associated with different biological functions such as sexual signaling, crypsis, and thermoregulation (Bond 2007). For land snails, crypticity and scape from predators may be the main advantages of color and color-band polymorphisms (Surmacki et al. 2013). Moreover, polymorphism can lead the number of species to be underestimated (Köhler and Burghardt 2016; Inkhavilay et al. 2017). Alternatively, on the other hand, it can also lead to the overestimation of diversity, in which individuals of the same species are described as belonging to distinct species, such as *L.carnavalescus*.

As we mentioned above, the species now ascribed to *Leiostracus* were allocated to different genera of Orthalicoidea in the past (Albers and Martens 1860; Pilsbry 1899; Ancey 1901; Breure 1979; Schileyko 1999), and these taxonomic attributions relied solely on the shell morphology. However, there is enough evidence of the separation of *Leiostracus* from other genera, which is supported by both shell and anatomical characters (Breure 1978; Schileyko 1999). *Leiostracus* can be distinguished from *Bulimulus* by its elongated shell and protoconch sculpture with wavy, or radial wrinkles, sometimes with granulations or reticulate on the lower part, while the latter exhibits regular oblique striation on the upper part and spiral irregular corrugate lines. Despite some superficial similarities, the shells of *Drymaeus* and *Leiostracus* can be clearly separated. According to Breure (1979), in *Leiostracus*, the shell is more conical, sometimes presenting a keeled or angled body whorl, with a higher spire and 7–8 whorls, while *Drymaeus* presents an oval shell shape, with a lower spire, and 5–6 whorls. In *Drymaeus*, the protoconch shows a reticulate sculpture, whereas the protoconch in *Leiostracus* has spiral striations. Considering the pallialsystem, the conspicuity of the pulmonary vein and secondary vessels is distinct in *Leiostracus* and the other genera. In species of *Leiostracus*, the pulmonary vein is well developed, and secondary vessels are moderately developed. In species of *Bulimulus*, the pulmonary vein and the secondary vessels are strongly developed, while *Drymaeus* species show veins weakly to moderately developed with the adrenal ureter partly open or veins strongly developed and the adrenal ureter varying from open to closed. Some differences are also found in the genital apparatus of these genera. In *Leiostracus*, the penis sheath is absent, while in *Drymaeus*, the absence or presence of a penis sheath can be observed in different species. In *Bulimulus*, the penis is swollen in the distal part and usually covered by a proximal sheath (Breure 1979).

*Leiostracusobliquus* differs from all other species of the genus due to its conical-oval shell, with a brown line on the base of the body whorl, a reflected apertural lip and by presenting a fine brown sutural line, which is an uncommon trait among species of this genus. This species can be distinct from *L.cinnamomeolineatus* due to the presence of spiral brown lines and the absence of a sutural line in the shell. Shell dimension is also different, with the shell of *L.obliquus* being slightly larger (~ 23 mm, with 7–8 whorls) compared to the shell of *L.cinnamomeolineatus* (~ 21.5 mm, with 6 2/3 whorls).

Our findings suggest that *Leiostracusobliquus* may be endemic to Brazil, where it is primarily found in Atlantic Forest areas. In the original description, Reeve (1849), mentioned the type locality was the state of Bahia, without giving a specific location where the material was found. The geographical distribution of this species remained largely unknown as subsequent authors (Pilsbry 1899; Ancey 1901; Breure 1978) mentioned records for the states of Minas Gerais, Bahia, and Espírito Santo, without reference to the specific localities. Dohrn (1883), mentioned that the material analyzed by him was collected in the headwaters of the Mucury River, eastern Minas Gerais, which corresponds to the locality mentioned in the original label of the material ZMA 385621, i.e., “Mucury, Brasilis”. Herein, most of the occurrence records of. *L.obliquus* associated with malacological collections corresponded to localities in the Southeast of Brazil. According to Simone (2006), the genus *Leiostracus* occurs in Bahia and Minas Gerais states. Other species also occur in the Southeast, with scarce records for Espírito Santo, Rio de Janeiro, and São Paulo states. In this regard, the compilation of occurrence records for *L.obliquus* made in this work, extends the known distribution of this genus in Brazil. According to our results, most of the records of *L.obliquus* corresponded to the Atlantic Forest, one of the most threatened biomes in South America, with more than 85% of its original area deforested (Ribeiro et al. 2009). Land snails have a limited potential for dispersal and rely on their surroundings to survive and reproduce (Baur and Baur 1988; Barahona-Segovia et al. 2019). Thus, they are among the most threatened animal groups globally. In Brazil, the scarcity of information on taxonomy, ecology, and distribution of native species of land snails, hampers the efforts in determining their conservation status and, consequently, to foment legal protection measures for land snail species occurring in the Atlantic Forest, one of the most threatened biomes and with more than 85% of the original area deforested, threatening many plant and animal species with extinction.

## Supplementary Material

XML Treatment for Leiostracus

XML Treatment for Leiostracus obliquus

## ﻿Additional information

### Author ORCIDs

Maria Isabel P. F. Macedo https://orcid.org/0000-0001-9272-0432

Ximena M. C. Ovando https://orcid.org/0000-0003-4541-3151

Sthefane D’ávila https://orcid.org/0000-0001-6494-309X

### Data availability

All of the data that support the findings of this study are available in the main text or Supplementary Information.

## Supplementary materials

Supplementary material 1 Localities of occurrences of *Leiostracusobliquus* in Brazil Data type table (docx. file) https://binary.pensoft.net/file/865907This dataset is made available under the Open Database License (http://opendatacommons.org/licenses/odbl/1.0/). The Open Database License (ODbL) is a license agreement intended to allow users to freely share, modify, and use this Dataset while maintaining this same freedom for others, provided that the original source and author(s) are credited.Maria Isabel P. F. Macedo, Ximena M. C. Ovando, Sthefane D’ávila
