# Supplementary material for: ﻿Redescription and geographical distribution of Leiostracusobliquus (Reeve, 1849) (Mollusca, Gastropoda, Simpulopsidae)
Source: Zookeys. 2023 Jun 19;1167:223–40. doi: 10.3897/zookeys.1167.98707 (PMC10293901; doi:10.3897/zookeys.1167.98707)
Supplement: Supplementary material 1 — Localities of occurrences of Leiostracusobliquus in Brazil [file zookeys-1167-223_article-98707__-s001.docx]

| State | Locality | Longitude | Latitude |
| --- | --- | --- | --- |
| Bahia | Itapetininga | -40.25622 | -15.252321 |
| Espírito Santo | Santa Teresa | -19.959258 | -19.959258 |
| Espírito Santo | Cachoeiro do Itapemirim | -41.158682 | -20.804452 |
| Espírito Santo | Linhares | -40.058149 | -19.393643 |
| Espírito Santo | Itaguaçu | -42.981312 | -14.322636 |
| Espírito Santo | Baixa Grande | -42.059626 | -22.858324 |
| Espírito Santo | Baixo Guandú | -40.94437 | -19.557108 |
| Espírito Santo | Pedro Canário | -40.013676 | -18.185076 |
| Espírito Santo | Itapemirim | -40.783757 | -20.902508 |
| Espírito Santo | Meaípe | -40.55 | -20.733333 |
| Minas Gerais | Mantena | -40.981338 | -18.783376 |
| Minas Gerais | Nanuque | -40.345326 | -17.842428 |
| Minas Gerais | Governador Valadares | -41.95719 | -18.855833 |
| Minas Gerais | Resplendor | -41.258085 | -19.327647 |
| Minas Gerais | Teófilo Otoni | -41.498154 | -17.876243 |
| Rio de Janeiro | Rio de Janeiro | -43.172758 | -22.906841 |

Table S1. Localities of occurrences of *Leiostracus obliquus* in Brazil.
